# A New *de novo* Mosaic Mutation of PHEX Gene: A Case Report of a Boy with Hypophosphatemic Rickets

**DOI:** 10.2174/1871530323666230227142202

**Published:** 2023-07-19

**Authors:** Alessandra Terracciano, Margherita Lucia De Bernardi, Roberto Novizio, Davide De Brasi, Achille Iolascon, Matteo Della Monica, Francesco Scavuzzo, Domenico Serino, Antonio Novelli, Carmelo Piscopo

**Affiliations:** 1 Translational Cytogenomics Research Unit, Bambino Gesù Children's Hospital, IRCCS, Rome, Italy;; 2 Department of Molecular Medicine and Medical Biotechnology, University Federico II, Naples, Italy;; 3 Endocrinology Unit, Cardarelli Hospital, Naples, Italy;; 4 Medical and Laboratory Genetic Unit, Cardarelli Hospital, Naples, Italy

**Keywords:** X-linked hypophosphatemia, vitamin D resistant rickets, dominant X-linked inheritance, phosphate regulating gene, FGF23, endopeptidase

## Abstract

**Background:**

X-linked hypophosphatemia is the most prevalent form of heritable rickets, characterized by an X-linked dominant inheritance pattern. The genetic basis of X-linked hypophosphatemia is a loss-of-function mutation in the PHEX gene (Phosphate regulating gene with Homology to Endopeptidases on the X chromosome), which leads to an enhanced production of phosphaturic hormone FGF23. X-linked hypophosphatemia causes rickets in children and osteomalacia in adults. Clinical manifestations are numerous and variable, including slowdown in growth, swing-through gait and progressive tibial bowing, related to skeletal and extraskeletal actions of FGF23. PHEX gene spans over 220 kb and consists of 22 exons. To date, hereditary and sporadic mutations are known (missense, nonsense, deletions and splice site mutations).

**Case Presentation:**

Herein, we describe a male patient carrying a novel *de novo* mosaic nonsense mutation c.2176G>T (p.Glu726Ter) located in exon 22 of PHEX gene.

**Conclusion:**

We highlight this new mutation among possible causative of X-linked hypophosphatemia and suggest that mosaicism of PHEX mutations is not so uncommon and should be excluded in diagnostic workflow of heritable rickets both in male and female patients.

## 1 INTRODUCTION

X-Linked Hypophosphatemia (XLH) is the most prevalent form of heritable rickets, with an approximate incidence of 4-5 cases per 100,000 persons, and a dominant inheritance pattern [1]. The genetic basis of XLH is a loss-of-function mutation in the PHEX gene (Phosphate regulating gene with Homology to Endopeptidases on the X chromosome) located at Xp22.1-p22.2 [2], which leads, through a not fully understood mechanism, to enhanced production of the phosphaturic hormone FGF23 by osteocytes as primary source, determining a selective renal phosphate wasting. PHEX gene (RefSeq NM_000444.6) spans over 220 kb and consists of 22 exons. It encodes the PHEX protein (RefSeq NP_000435.3), belonging to the zinc binding endopeptidase family with an amino acid similarity to neutral endopeptidase (NEP) accounting to approximately 60%. The protein has a short 20 amino acids intracytoplasmic tail, a 25 amino acids transmembrane region and a long 704 amino acids ectodomain. The catalytic site characterizing the endopeptidase family is encoded in exon 17 [3, 4]. Consensus motifs, containing substrate interacting residues, and the binding zinc site have been identified in exons 15 and 19 respectively [5]. To date, familial and sporadic cases are described, associated with missense, nonsense, deletions and splice site mutations. Up to now, 51.2% of missense mutations identified were located in mentioned exons [6]. Although based on an X-linked inheritance pattern, assuming then that males should have a more severe phenotype than females, there are no significant sex differences in clinical or biochemical parameters. A stronger correlation seems to exist with genomic variant and location [7]. Herein, we describe a male patient with a novel *de novo* mosaic nonsense mutation c.2176G>T (p.Glu726Ter) located in exon 22 of PHEX gene.

This case report was prepared following the CARE guidelines [8].

## 2 CASE REPORT

Proband is the first of two male siblings of healthy, caucasian, nonconsanguineous parents. He was born after a caesarean section at the 41^st^ gestational week, after an early threat of abortion occurring at the 22^nd^ gestational week. During pregnancy, a univentricular heart with transposition of the great vessels was detected and then postnatally confirmed. His birth weight was 3,750 g (50^th^ -75^th^ centile), birth height was 50 cm (50^th^ centile), head circumference was 34.5 cm (50^th^ centile), and Apgar scores were 7-7 (1^st^ and 5^th^ minutes). Three corrective heart surgeries were performed during infancy: at 2 months of age, patient underwent pulmonary artery banding and stenting of the arterial ductus; at 2 years old he underwent Glenn bidirectional cavopulmonary shunt procedure; at 7 years old he underwent total cavo-pulmonary connection. Moreover, a small ventricular septal defect and a mild aortic valve regurgitation required further pharmacological and surgical procedures. During growth, psychomotor development and language learning were normal, with walking-alone starting at 15 months.

At the nineteenth month of life, because of a progressive slowdown in growth, swing-through gait and progressive tibial bowing, the patient underwent x-ray evaluation of the lower limbs, confirming bowing of the tibial shaft and revealing the widening of distal femoral metaphysis.

At the thirty-fourth month of life, his height and body weight were 89 cm (13^th^ centile; -1.13 SD) and 14 kg (50^th^ centile), respectively. Bone age corresponded to biological age. The calculated target height for the child was 167.5 cm (14^th^ centile, -1.08 SD). A careful inquiry into the patient’ family history didn’t reveal any relevant information.

During clinical evaluation, some laboratory tests were performed, showing serum hypophosphatemia (3 mg/dL; normal range, 3.2-6.5 mg/dL), normal serum calcium level (9.5 mg/dL; normal range: 8.8-10.8 mg/dL), normal serum creatinine (0.6 mg/dL; normal range, 0.6-0.9 mg/dL), and increased serum alkaline phosphatase (902 IU/L; normal range, 30-525 IU/L). The intact parathyroid hormone level was 113 pg/mL (normal range, 10-80 pg/mL); 25-hydroxyvitamin D level was 24.7 ng/mL (normal range: 20-55 ng/mL). The urine tests showed: calcium/creatinine ratio, 0.18 mg/mg (normal range: 0.01-0.24 mg/mg); phosphate/creatinine ratio 1.18 mg/mg (normal range: 0.22-0.86 mg/mg). The tubular reabsorption of phosphate (TRP) measured in 24-hour urine sample was reduced to 77% (normal range over 85%). The tubular threshold of phosphate (TmPO_4_) per volume unit of glomerular filtration rate (TmPO_4_/GFR) calculated using the Walton & Bi-jvoet nomogram was 2.4 mg/dL (normal range: 2.9-6.5 mg/dL) (Table **1**). A neck ultrasound study showed mild right parathyroids hyperplasia. Nutritional, hepatic, renal, lipidic, celiac, thyroid profiles and serum somatomedin levels were all normal. No proteinuria and no glycosuria in the urine exam. Clinical teeth examination, audiometric test and abdominal ultrasound were normal as well. Based on the above results, diagnosis of hypophosphatemic vitamin D-resistant rickets was supposed.

At the age of 3 years, calcitriol (starting dose: 0.25 µg/day increased on time to 0.5 µg twice a day) was started, and an oral phosphate supplementation (Joulie’s solution - 30.4 mg of phosphorus/mL, starting dose: 36 mg/Kg/day of elemental phosphorus increased on time according to body weight) was added without success, but progressive worsening of varus limbs, slowing in growth rate (4.0 cm/year < 3^th^ centile in the third year of treatment) and persistent hypophosphatemia was objectified.

Three years later, at the age of 6, Joulie’s solution was stopped and replaced by another oral phosphate supplementation, Reducto Spezial (613 mg/tablet, four times a day), associated with calcitriol (0.5 µg/tablet, twice a day). After two years of this therapy, when the patient was 8 years and six months old, his height and body weight were 118 cm (3^rd^ centile -1.85 SD) and 42 kg (> 97^th^ centile), respectively. His linear growth rate was increased (5.5 cm/year in the first and 5.7 cm/year in the second year of treatment) and alkaline phosphatase, serum phosphate and parathyroid hormone levels returned to normal ranges. At that time an x-ray evaluation of the lower limbs highlighted the varus state and the metaphyseal bowing of the femur and tibia, with wrist bone age corresponding to chronological age according to Greulich & Pyle atlas.

At the age of 11 years, he underwent a knee CT scan that revealed a deformity of the femoral condyles and tibial plates, with degeneration and thinning of lateral menisci.

During the growth, auxologic parameters constantly showed short stature (<3^rd^ centile), low height velocity (<25^th^ centile) and severe obesity in pediatric and pubertal ages. He underwent normal pubertal development, but no real growth spurt was observed.

**Fig. (1):**
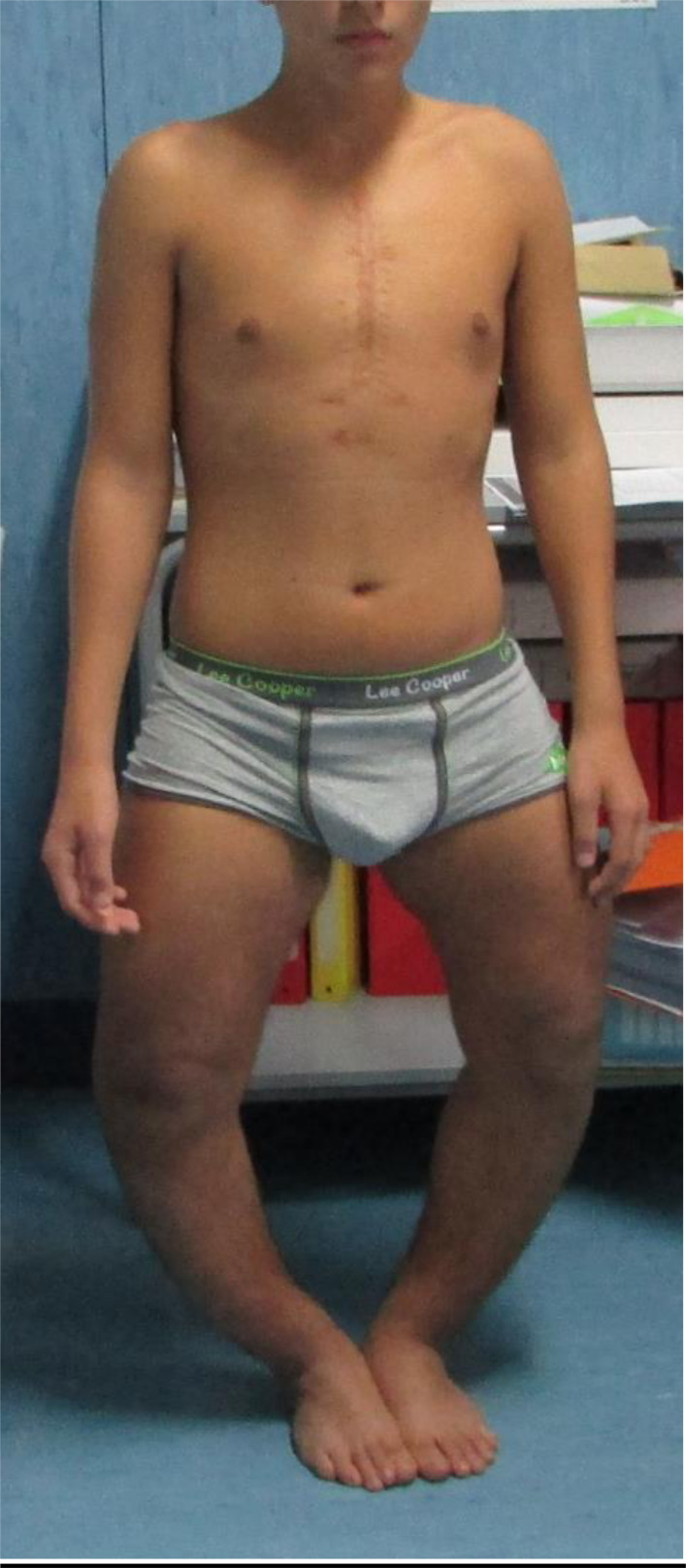
Severe genu varum, tibial varism.

**Fig. (2):**
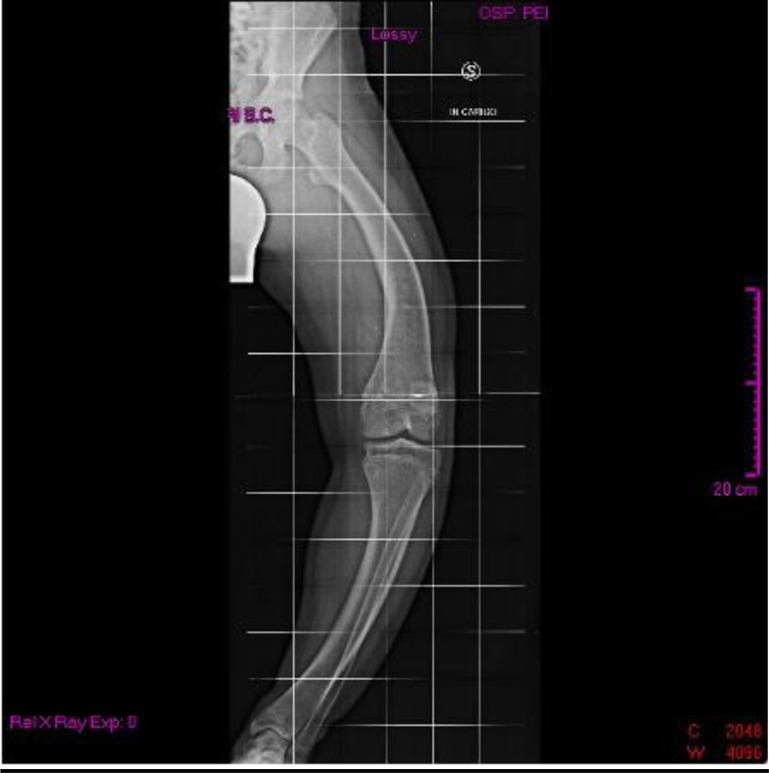
X-ray evaluation of the left leg.

**Fig. (3):**
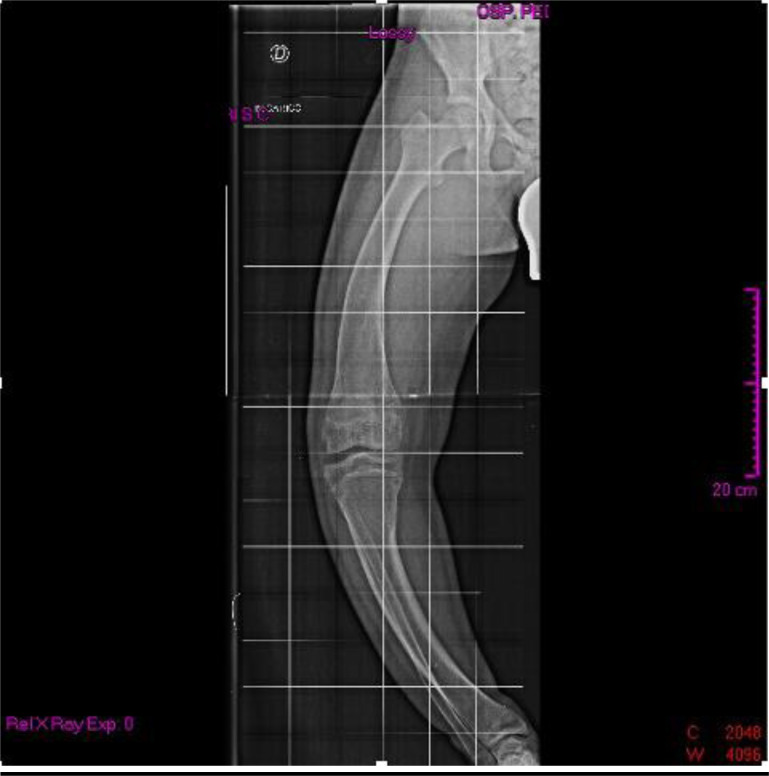
X-ray evaluation of the right leg.

When he was 15 years old, he was admitted to our Clinical Genetic Unit. Physical examination showed the following parameters: height 150 cm (<3^rd^ centile, -2.2 SDS), weight 63.5 kg (75^th^ - 90^th^ centile), arm span 167 cm, SPAN/Height ratio: 1.11, occipito-frontal circumference 55 cm (50^th^ centile). Pubertal development was normal (P4 G4 according to Tanner stages). The clinical exam revealed a long face with mild bitemporal constriction, severe genu varum, tibial varism and plantar supination during walking (Fig. **1**). X-ray evaluation of the legs confirmed the clinical findings (Figs. **2** and **3**).

At 16 years old, a Bone Densitometry (DEXA) of the lumbar spine and left femur was performed, revealing femoral osteopenia (t-score: - 1.2 SD). At the same age, a neck ultrasound showed normal thyroid parenchyma, without any pathological parathyroid findings. Up to now, patient is still on phosphate supplementation (Reducto Spezial 613 mg/tablet, five times a day) and calcitriol therapy (0.5 tablet, twice a day), maintaining serum calcium, alkaline phosphatase, intact parathyroid hormone and 25-hydroxyvitamin D levels within the normal range and serum phosphorus at the lower limit of the normal range. During years of medical treatment, therapy adherence and tolerability have always been referred to be high, with no relevant adverse effects. Currently, the patient is on orthopedic, cardiological and endocrinological follow-up, waiting for a surgical correction of limb deformity.

## 3 GENETICS ANALYSES

Karyotype performed postnatally revealed a normal male pattern, with FISH analysis negative for del22q11.2Array-Comparative Genomic Hybridization showed normal arr(1-22)x2,(X,Y)x1. The molecular basis of the disease was identified for the proband with next-generation sequencing technology, using the Sophia Genetics Nephropathies Solution panel, covering 44 genes clinically relevant to kidney diseases on the Illumina platform (NextSeq550). The variant c.2176G>T (p.Glu726Ter) in the exon 22 of the PHEX gene was identified on DNA extracted from a blood sample in an apparent heterozygous state (47% VAF variant allele frequency). Given the PHEX gene localization on chromosome X and the male patient, a mosaicism was suspected. Alignment, quality filtering, variant calling and variant annotation were performed using the SOPHiA DDM^TM^ platform version 5.10.21 pipeline (SOPHiA GENETICS SA, Switzerland). The variant calling files were filtered using SOPHiA DDM^TM^ platform and the detected variants were characterized according to the recommendations of the American College of Medical Genetics and Genomics (ACMG) [9]. The variant was novel, never reported in ClinVar and gnomAD and characterized as pathogenic according to ACMG criteria. It was absent from the population databases of the Exome Sequencing Project and Genome Aggregation Database (PM2 criterion) and pathogenicity predictions were all in favor of a damaging effect of this amino acid substitution (PP3 criterion) (SIFT: 0.000, PolyPhen-2 HumVar: 0.995, Mutation Taster: 1.000 and DANN: 0.9977). The nonsense variant was located in the C-terminal region leading to a truncated protein lacking the last 23 amino acids. Moreover, other loss of function genomic variants is already described in the same exon 22. Sanger sequencing was used to test the PHEX genomic variant on the blood of the patient’s parents and brother, as well as on a second tissue’s DNA (saliva) of the proband. The technique confirmed the *de novo* origin of the mutation as well as its mosaic condition. Quantization of the gene copies of PHEX of the proband was completed by qPCR assay with ABI 7900HT (ABI, Los Angeles, CA, USA) Real-time PCR System. Two normal males and two females were taken into the assay as controls. β-Globin gene was used as an internal reference gene to confirm the same amount of gene copies of PHEX as normal males. SYBR Green was applied as a nucleic acid stain (Power SYBR™ Green PCR Master Mix, ThermoFisher).

## 4 DISCUSSION

Hereditary hypophosphatemic rickets is a group of genetically heterogeneous disorders, consisting of X-linked dominant hypophosphatemic rickets caused by a mutation in PHEX gene, autosomal dominant hypophosphatemic rickets caused by a mutation in FGF23, autosomal recessive hypophosphatemic rickets caused by a mutation in DMP1, ENPP1, FAM20C or SLC34A3 and X-linked recessive hypophosphatemic rickets caused by a mutation in CLCN5. In the case here described, the patient's parents were healthy, so all molecular forms were possible and the genetic diagnosis was paramount. Given the high penetrance of XLH in carrier women, since the mother and the brother were phenotypically normal and not hypophosphatemic (a normal TRP was calculated in both the parents and in the brother), the presence of a *de novo* mutation was supposed. Genetic tests confirmed our hypothesis because they identified a novel *de novo* nonsense mutation in PHEX gene c. 2176G>T in the last exon 22 both on DNA extracted from peripheral leukocytes and on DNA extracted from a second tissue (saliva). Even if further analysis on protein function is missing, we can speculate about a Loss of Function (LOF) effect of C-terminal truncation. This truncating effect involves both the longer and shorter forms of the transcript, considering that both include the last exon in the splicing process. Since we first receive the patient at the age of fifteen, most information about his medical history has been obtained through anamnesis and medical reports, with possible bias that could constitute a limitation.

Generally, *de novo* mutations arise in the sperm or egg of one parent or a fertilized egg. Once inherited, they will be present in all cells. In this patient, the mosaic pattern for a mutation appeared probably in the early postzygotic period, so that mutation is present in most but not all tissues.

## CONCLUSION

This is the third reported PHEX mosaic male in literature. All the mosaic mutations described up to now, two splicing variants [10, 11] and the nonsense c.2176G>T here described, seem to lead to truncated PHEX protein losing its active site, Zn^2+^-binding site and four conserved cytosines, which probably undergoes loss-of-function and structural instability. PHEX protein is essential in bone and dentin mineralization and renal phosphate reabsorption, thus the loss-of-function of PHEX is probably the cause of the low phosphate concentration and the abnormal bone mineral density.

Through segregation analysis, we demonstrated the *de novo* origin of the variant. A second tissue (saliva) analysis other than DNA extracted from the blood sample, confirmed the somatic mosaic condition.

We also demonstrated that there was no duplication of the PHEX gene that occurred on the X chromosome because the copy number of the gene was identical to normal males based on qPCR.

## AUTHORS’ CONTRIBUTIONS

All authors have equally contributed to the clinical follow-up of the patient and the preparation of this manuscript. All authors contributed to the article and approved the submitted version

## Figures and Tables

**Table 1:** Blood and urine tests summary.

**Blood Profile**	**Value**	**Reference Range**
Phosphate	3 mg/dL	3.2-6.5 mg/dL
Total calcium	9.5 mg/dL	8.8 - 10.8 mg/dL
Serum creatinine	0.6 mg/dL	0.6 - 0.8 mg/dL
Serum alkaline phosphatase	902 IU/L	30 - 525 IU/L
Parathyroid hormone	113 pg/mL	10 - 80 pg/mL
25-hydroxyvitamin D3	24.7 ng/mL	20 - 55 ng/mL
**Urine Profile**		
Calcium/creatinine ratio	0.18 mg/mg	0.01 - 0.24 mg/mg
Phosphate/creatinine ratio	1.18 mg/mg	0.22 - 0.86 mg/mg
Tubular reabsorption of phosphate (TRP)	77%	> 80%
Tubular threshold of phosphate per volume unit of glomerular filtration rate (TmPO4/GFR)	2.4 mg/dL	2.9 - 6.5 mg/dL

## Data Availability

Not applicable.

## LIST OF ABBREVIATIONS

ACMG: American College of Medical Genetics and Genomics
LOF: Loss of Function
NEP: Neutral Endopeptidase
